# Proton or photon? Comparison of survival and toxicity of two radiotherapy modalities among pediatric brain cancer patients: A systematic review and meta-analysis

**DOI:** 10.1371/journal.pone.0318194

**Published:** 2025-02-20

**Authors:** Renáta Kiss-Miki, Mahmoud Obeidat, Vanda Máté, Brigitta Teutsch, Gergely Agócs, Szilvia Kiss-Dala, Péter Hegyi, Janka Kovács, Andrea Párniczky, Eszter Tuboly, Miklós Garami

**Affiliations:** 1 Centre for Translational Medicine, Semmelweis University, Budapest, Hungary; 2 Neurosurgery and Neurointerventional Clinic, Semmelweis University, Budapest, Hungary; 3 Pediatric Center, Semmelweis University, Budapest, Hungary; 4 Institute for Translational Medicine, Medical School, University of Pécs, Pécs, Hungary; 5 Institute of Pancreatic Diseases, Semmelweis University, Budapest, Hungary; 6 Heim Pál National Pediatric Institute, Budapest, Hungary; 7 Hungarian Pediatric Oncology Network, Budapest, Hungary; Central Research Institute of Electric Power Industry (CRIEPI), JAPAN

## Abstract

**Background:**

With the introduction of new therapy modalities and the resulting increase in survival rates, childhood brain cancers have become a focal point of research in pediatric oncology. In current protocols, besides surgical resection and chemotherapy, radiotherapy is required to ensure optimal survival. Our aim was to determine which of the two major irradiation options, proton (PT) or photon (XRT), was the least harmful yet effective for children with brain tumors.

**Methods:**

The protocol was registered on PROSPERO in advance (CRD42022374443). A systematic search was performed in four databases (MEDLINE via (PubMed), Embase, Cochrane Library, and Scopus) on 23 April 2024. Odd ratios (OR) and mean differences (MD) with 95% confidence intervals (CI) were calculated using a random-effects model. Survival and six major types of side effects were assessed based on data in the articles and reported using the Common Terminology Criteria for Adverse Events (CTCAE) version 5.0. Heterogeneity was assessed using Higgins and Thompson’s I^2^ statistics.

**Results:**

Altogether, 5848 articles were screened, of which 33 were eligible for data extraction. The 5-year overall survival results showed statistically no significant difference between the two radiotherapy modalities (OR = 0.80, 95% CI: 0.51–1.23, *p* = 0.22, *I*^*2*^ = 0%). In terms of toxicity rates, an advantage was found for PT, particularly in terms of chronic endocrine side effects (hypothyroidism OR: 0.22, 95% CI: 0.10–0428, *p* = 0.002, *I*^*2*^ = 68%), neurocognitive decline (global IQ level MD: 13.06, 95% CI: 4.97–21.15, *p* = 0.009, *I*^*2*^ = 68%). As for hematological, acute side effects, neurological changes and ophthalmologic disorders PT can be beneficial for survivors in terms of reducing them.

**Conclusions:**

In comparison with XRT, PT can reduce most side effects, without significantly decreasing the survival rate. **T**here is considerable clinical relevance in the findings, even not all of them are statistically significant, which may facilitate the development of protocols regarding the usage of radiotherapy methods, and may encourage the establishment of more proton centers, where more studies can be done.

## Introduction

Tumors of the brain or central nervous system (CNS) represent a major challenge in pediatric oncology, with a high impact on the quality of life of these young patients. More than 4,000 CNS tumors are diagnosed in children every year [1]. With this number, this pathology represents a huge challenge for pediatric neuro-oncologists, but it is a burden for the family and the society as well.

In the last decades, there has been a huge development in the field of treatment, both in chemotherapy agents and in the radiotherapy part as well. Although effective in managing tumor growth [2,3], traditional radiation treatment methods are often associated with a number of side effects that can detrimentally affect the development and overall well-being of a child [3]. The need for a more targeted and less harmful treatment approach is imperative. Proton radiotherapy, with its unique dose characteristics, has recently emerged as a promising solution to this problem [4–6].

Causes of childhood brain cancers vary, and besides genetic factors, numerous epigenetic factors could be identified, such as low birth weight [5,7], parental lead exposure [8], non-chromosomal structural birth defects, or higher socioeconomic position [9].

Besides chemotherapy and surgery, radiotherapy plays an important role in treatment protocols. Two major radiotherapy modalities can be identified: the oldest is photon therapy (XRT), which has undergone significant development in the last decades. Intensity-modulated therapy (IMRT), stereotactic radiosurgery, and stereotactic body radiation therapy (SBRT) are promising techniques [10,11]. The newest radiotherapy method (IMRT) was developed with many promising outcomes by reducing the toxicity, but may also lead to adverse events by triggering different biological processes [12].

On the other hand, proton therapy (PT) represents a new and significant advancement in the treatment of pediatric brain tumors. Despite the fact that the radiobiological effect is similar between proton and photon therapy, there is a robust difference considering healthy tissue sparing [13]. As technology continues to advance and more clinical research is conducted, PT is expected to become an even more integral part of pediatric oncology.

Children receiving radiotherapy are at risk of neurocognitive decline, neuroendocrine dysfunction, hearing loss, vascular anomalies, psychosocial dysfunction [2,14,15], secondary cancer development [2,16], and acute toxicities (e.g., nausea, vomiting, fatigue) [2,17]. The risk of these side effects correlates with the area irradiated [16].

Currently, there is no complex and comprehensive consensus (evidence-based) in the literature regarding the therapeutic recommendations for XRT and PT in childhood brain tumors. This study aims to compare the efficacy and safety of XRT with PT in the treatment of pediatric brain cancer patients, drawing on a systematic review and meta-analysis of existing literature.

Our hypothesis was that PT modalities would be more effective and less harmful for pediatric brain cancer patients compared to XRT.

## Methods

We report our systematic review and meta-analysis based on the Preferred Reporting Items for Systematic Reviews and Meta-Analyses (PRISMA) 2020 guidelines [18] (S1 Table in S1 File); the recommendations of the Cochrane Handbook were also followed [19]. The protocol was registered on PROSPERO (CRD42022374443) in advance, and we fully adhered to it; however, some changes were necessary because of the statistical analysis of data in the articles.

### Ethical approval

No ethical approval was required for this systematic review with meta-analysis, as all data were already published in peer-reviewed journals. No patients were involved in the design, conduct, or interpretation of our study.

The datasets used in this study can be found in the full-text articles included in the systematic review and meta-analysis.

### Eligibility criteria

We included studies of children with brain tumors who received XRT or PT and reported side effects based on Common Terminology Criteria for Adverse Events (CTCAE) version 5.0 or data on overall survival (numerical data or Kaplan Meier curves). All comparative study types and abstracts were also included in the analysis, in which both XRT and PT were investigated by the same working group and enough amount of information was provided regarding the two modalities and outcomes. Articles investigating only one radiotherapy modality or describing dose comparison or therapy modeling were ultimately excluded, such as articles from where data were missing regarding the measured outcomes, or they were reported in a non-reproducible way.

We used the PICO framework (population, intervention, comparison, outcome) [20] to define our eligibility criteria. We investigated PT as an intervention versus XRT as a comparator in the population of children with brain tumors with our outcomes, including survival, neurocognitive decline, hematological, endocrine, neurological, ophthalmologic, acute and other side effects.

### Information sources

Our systematic search was conducted on 25 November 2022 and updated on 23 April 2024 in four databases: MEDLINE via PubMed (n = 1400), Embase (n = 1890), Cochrane Library (n = 9), and Scopus (n = 653 with title abstract selection). No restrictions were applied. Additionally, backward and forward citation searching was conducted after completion of the full-text selection to identify other potentially relevant publications.

### Search strategy

Our search key consists of four main domains: pediatric population, brain cancer, photon, and proton radiotherapies. For the detailed search strategy, see S2 Table in S1 File.

### Screening and selection

After the systematic search, we imported the articles into the reference management system (EndNote 20.1). Duplicate articles were eliminated automatically and manually based on overlapping years, authors, and titles. Screening and selection were done by two independent reviewers (R.K-M and J.K), first by title-abstract selection and then by full-text selection. Cohen’s kappa was calculated at all levels of selection. In case of disagreement, consensus was reached after discussion with the corresponding author (M.G). No automation tools were used in the selection process.

### Data extraction

Data from the eligible articles were collected independently by two authors (R.K-M and J.K). Disagreements were resolved by involving the corresponding author (M.G). All data were collected manually and entered an Excel spreadsheet (Office 365, Microsoft, Redmond, WA, USA) for analysis. During data extraction, we identified conference abstracts and articles with the same population, one of which had the largest sample size or the most reported side event outcomes.

### Data items

The following data were extracted: first author, year of publication, study population, study period and patient data and demographics (e.g., number of patients included, tumor type, age), dose of radiotherapy, follow-up period and outcomes investigated: endocrine deficit (hypothyroidism, growth hormone, sex hormone and adrenal deficiency), ototoxicity, neurocognitive decline outcomes (intelligence quotient (IQ), verbal reasoning, perceptual reasoning, working memory, processing speed), hematological outcomes (anemia, leukopenia, lymphopenia, thrombocytopenia, neutropenia), acute side effects (nausea, vomiting, ophthalmic disorder, neurological disorder, skin disorders, fatigue, headache, diarrhea, constipation, anorexia, insomnia, esophagitis, abdominal pain) and others (body mass index, obesity, vascular injury, ventriculo-peritoneal shunt placement).

Outcomes were reported in most of the articles using the CTC-AE scale. For hematological outcomes, we used grade 3 and 4 toxicity.

Neurocognitive domains in the articles [21–25] were measured using different age adapted questionnaires, such as the Wechsler Intelligence Scale IV and V, the Woodcock-Johnson Test of Cognitive Abilities, and the Stanford-Binet Intelligence Test.

Overall survival was analyzed using raw data reported in the articles and the Kaplan-Meier curves using a plot analyzer (software or website).

### Risk of bias assessment and quality of evidence

Two authors (R.K-M, J.K) independently assessed the risk of bias using the Risk of Bias in Non-randomised Studies-Intervention (ROBINS-I tool) [26]. To assess the quality of evidence of our results. We followed the Grading of Recommendations Assessment, Development and Evaluation (GRADE) [27] approach and used the GRADEpro tool (software). Study design, risk of bias, inconsistency, indirectness, and imprecision were the determinant factors.

### Synthesis methods

Statistical analysis was conducted using the statistical software R (version 4.1.2.) [28] and the *meta* package (version: 6.1.0) was used [29]. A minimum of three studies were required to perform statistical analysis. We used forest plots to summarize the findings of the studies and present the pooled result.

For dichotomous outcomes, pooled odds ratios (OR) were calculated with 95% confidence intervals (CI) using the Mantel-Haenszel method of random-effects [30]. Tau squared was estimated with the Paule-Mandel method [31].

For continuous outcomes, pooled mean differences (MD) were calculated with 95% CI using random-effects models and the inverse variance method. Tau squared was estimated using the restricted maximum-likelihood method [32].

In all cases, heterogeneity was examined by calculating the Higgins & Thompson I squared statistics [33] indicator and performing the Cochrane Q test [32]. Hartung-Knapp adjustments were also applied where necessary.

We performed a sensitivity analysis, including a leave-one-out influential analysis, to observe whether omitting one study was going to result in a considerable change in our results.

## Results

### Search and selection

**Fig 1:**
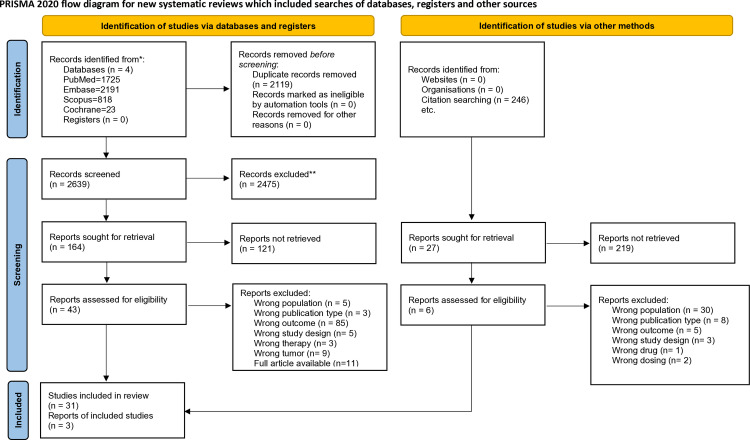
PRISMA flowchart of the article selection process.

Altogether, 5848 studies were identified using our search key in the four databases. After duplicate removal, we screened 2639 articles by title and abstract, and 73 articles were selected by full text, of which 54 were eligible for full text selection. Ten conference abstracts were excluded due to data being comprehensively reported elsewhere. In the end, 33 papers were eligible for data extraction. The selection process is presented in the PRISMA flowchart (Fig 1).

### Basic characteristics of studies included

Altogether, data from 2900 patients (1423 in the photon group and 1477 in the proton group) were collected from the articles and analyzed. The studies were conducted in the USA (mainly Texas), Spain, Germany, Denmark, United Kingdom and Korea. Baseline characteristics of the enrolled articles are presented in Table 1. Nine of the 33 studies were conference abstracts [34–41].

**Table 1:** Basic characteristics of the studies included.

Author (year)	Study site	Number of analyzed patients.	Age (year) min-max	Patients who received photon (nr.)	Patients who received proton (nr.)	Follow up period (months)	Total CSI dose (Gy) min-max	Total bed side dose (Gy) min-max	Tumor type
Paulino et al. 2018 [42]	Texas	84	2.9–18	46	38	–	18–39.60 CS axis	54.0–55.8	medulloblastoma
Paulino et al. 2021 [43]	Texas	115	3–17	63	52	–	18–39.60	54.0–55.8	medulloblastoma
Bielamowitz et al. 2018 [44]	Texas	95	2.15–16.2	54	41	–	23.4–39.60	36–59.4	medulloblastoma
Kahalley et al. 2019 [22]	Texas	79	3.55–14.85	42	37	0.10–10.9	15–39.6	51.0–59.4	medulloblastoma
Gross et al. 2019 [23]	Chicago	125	5.2–11.6	67	58		23.4–36.0	–	CNS tumors
Aldrich et al. 2021 [45]	Texas	118	2.4–21.6	54	64	12–120	15–39.60	54–55.8	medulloblastoma
Bishop et al. 2014 [46]	Texas	52	–	31	21	4.7–185.3	–	50.4–54.0	craniopharyngioma
Eaton et al. 2021 [21]	Massachusetts	88	3.4–20	20	17	12–136.8	18–27	54–>55.8	medulloblastoma
Yip et al. 2022 [47]	California	112	1.19–20	80	32	6–229.2	–	20–60	medulloblastoma, astrocytoma, ependymoma
Child et al. 2021 [24]	Texas	88	0.9–18	30	58	–	18–39.6	–	CNS tumors
Peterson et al. 2018 [48]	Petersburg	39	–	17	22	–	–	–	CNS tumors
Song et al. 2014 [49]	Korea	43	2–18	13	30	2–118	19.8–39.6	30.6–61.2	CNS tumors
Almutlaq et al. 2022 [50]	Indianapolis	78	–	25	45	–	23–39	.	medulloblastoma
Eaton et al. 2015 [51]	Massachusetts	105	3.3–21.9	60	45	–	18–27	54–>55.8	medulloblastoma
McElroy et al. 2018 [41]	Oklahoma	35	–	17	18	–	–	.	CNS tumors
Vatner et al. 2015 [39]	USA	170	–	52	118	–	18–36	49–72	medulloblastoma
Legault et al. 2013 [52]	New York	66	1.28–20.61	55	11	–	–	–	medulloblastoma
Okcu et al. 2022 [36]	Texas	102	–	44	58	–	–	–	medulloblastoma
Hopper et al. 2019 [35]	California	38	2–16	13	25	–	23.4–39.6	–	CNS tumors
Hong et al. 2020 [53]	Seul	126	4.15–22	93	32	0.25–14.15	19.8–36.0	16.2–45.0	CNS tumors
Uemura et al. 2022 [54]	Kobe	63	0.5–18.1	36	26	–	12–36	–	CNS tumors
Liu et al. 2021 [38]	Multiinstitutional	97	3.5–22.7	37	60	0.2–17.5	18–36	52.2–55.8	medulloblastoma
Warren et al. 2022 [25]	Texas	58	0.9–15–5	20	38	1.2–13.9	30.6–59.4	–	CNS tumors
Lassaletta et al. 2021 [55]	Madrid	8	–	4	4	–	36–39.6	55.8^a^	medulloblastoma
Eaton et al. 2014 [34]	Atlanta	105	3–21	60	45	0.6–19.70	18.0–37.2	16.2–37.8	medulloblastoma
Sato et al. 2017 [56]	Texas	79	0.4–18.7	38	41	7.2–140.4	–	54–59.4	CNS tumors
Weutschof et al. 2021 [57]	Germany	103	2.3–19	30	26	24–206.4	–	16–74	CNS tumors
Ravindra et al. 2021 [58]	Texas	63	5–16	14	18	–	–	50.4–54	craniopharyngioma
Sparber-Suaer et al.2023 [59]	Germany	397	0–21	91	127	5.3–23–4	–	32–50.5	parameningeal rhabdomyosarcoma
Baunsgaard et al. 2023 [60]	Denmark	41	0.4–14.6	30	11	–	12–61	–	Medulloblastoma,astrocytoma
Unnikirschnan et al. 2023 [61]	California	49	1.39–17.41	32	17	–	20–59.4	54^a^	CNS tumors
Mash et al. 2023 [62]	Texas	80	0.8–17.9	29	51	102–375.6	–	30.6–59.4	CNS tumors
Friedrich et al. 2023 [63]	United Kingdom	99	1.6–17.9	35	64	28.8–169.2	–	50–54	craniopharyngeoma

CNS: central nervous system, Gy: Gray, min: minimum, max: maximum

a: only the median value was reported

### Overall survival

**Fig 2:**
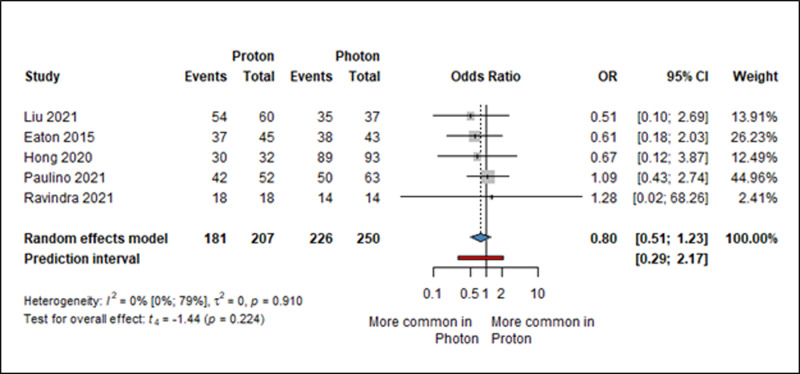
Forest plot showing the odds of 5-year overall-survival rate among children with brain tumors treated with either PT or XRT. OR: odds ratio, CI: confidence interval. Events means survival.

We found data on overall survival (three, five, ten years) in seven articles [38,43,46,51,53,56,58], in six of which Kaplan-Meier survival curves were also detected and analyzed. Survival data were available for five of the seven five-year, two of the articles for three years and one for 10 years. There was no statistically significant difference between the XRT and PT group for 5-year overall survival (OR: 0.80, 95% CI: 0.51–1.23, *I*^2^ = 0%, *p* = 0.224) (Fig 2). Data from the Kaplan-Meier curves for 3, 6, and 9-year survival can be found in the Supplementary Material, where survival probabilities were calculated, with no difference between the two radiotherapy modalities statistically (S1, S2, S3 Figs in S1 File).

### Endocrine side effects

**Fig 3:**
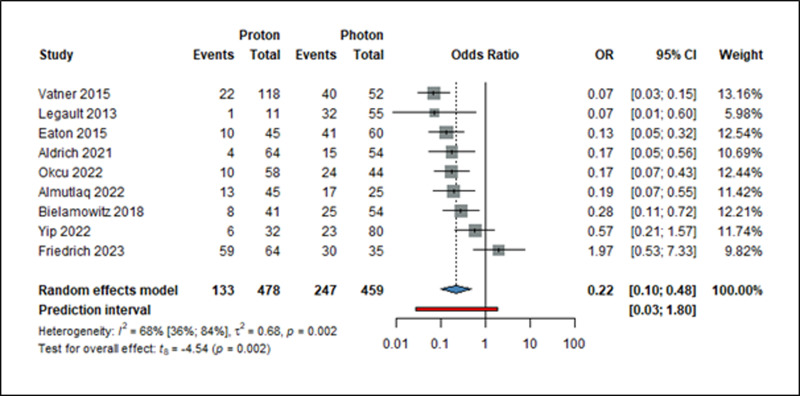
Forest plot showing the ratio of odds of hypothyroidism (=event) among pediatric brain cancer patients treated with either PT or XRT. OR: odds ratio (proton/photon), CI: confidence interval.

We analyzed the most frequent ones: hypothyroidism [21,36,39,44,45,47,50–52,60,63], growth hormone deficiency [39,47,51,60,63], and sex hormone deficiency [47,50,51,63]. A total of 937 patients were analyzed for hypothyroidism. In this cohort, the odds of developing hypothyroidism were almost five times higher with XRT than with PT(OR: 0.22; 95% CI: 0.10–0.48; *I*^*2*^ = 68%, p = 0.002) (Fig 3).

For growth hormone deficiency (OR: 0.69, 95% CI: 0.16–2.89, *I*^*2*^: 85%, *p* = 0.508) and sex hormone deficiency (OR: 0.39, 95% CI:0.09–1.63, *I*^*2*^ = 47%, *p =* 0.127), the number of patients was substantially lower, and the differences were not statistically significant (S4 and S5 Figs in S1 File).

### Neurocognitive side effects

**Fig 4:**
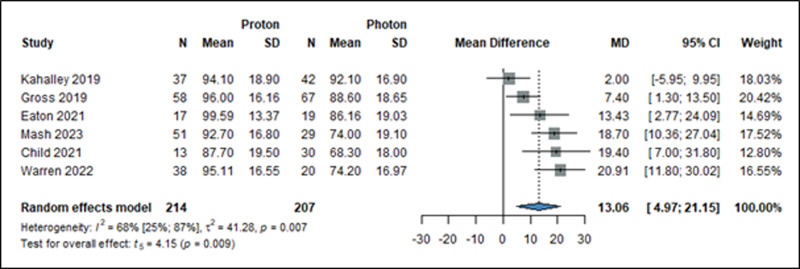
Forest plot showing the mean difference of global IQ level among pediatric brain cancer patients treated with either PT or XRT. MD: mean difference, CI: confidence interval.

Neurocognitive domains in the articles [21–25,61,62] were measured using different questionnaires, with mean scores as measures of effect. In terms of IQ level changes (MD: 13.06; 95% CI: 4.97–21.15; *I*^*2*^: 68%, *p*: 0.009), higher IQ scores were observed when patients received PT (Fig 4). Patients receiving PT performed better in terms of working memory (MD: 6.99, 95% CI: −4.10 - 18.08, *I*^*2*^: 74%, p: 0.155), processing speed (MD: 7.58, 95% CI: 0.37–14.78, *I*^*2*^: 68%, p:0.042), and perceptual reasoning (MD: 10.51 95% CI: −0.43 to 21.45, *I*^*2*^: 57%, p: 0.055) compared to patients receiving XRT (S6, S7, and S8 Figs in S1 File).

### Acute side effects and others

**Fig 5:**
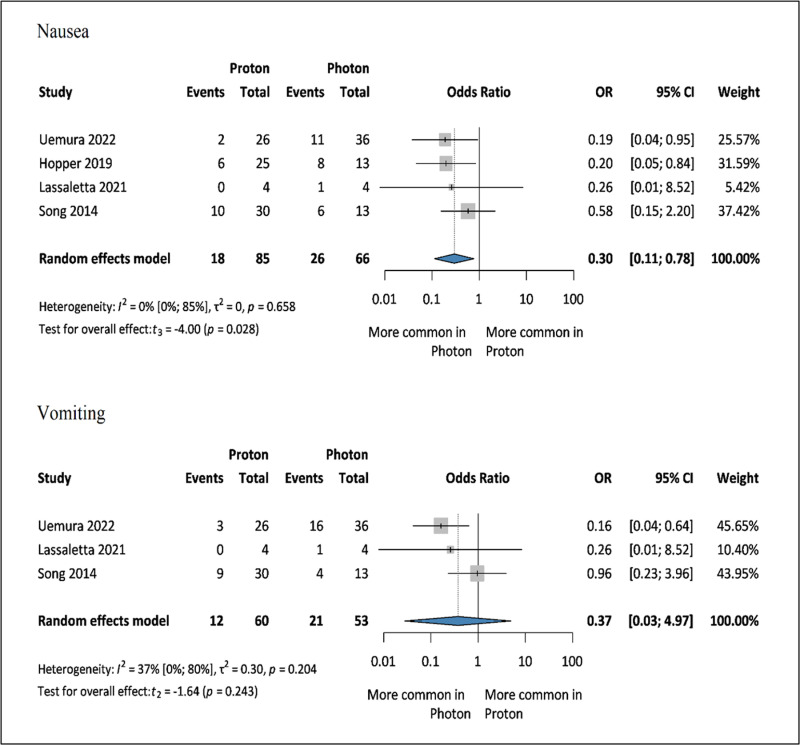
Forest plot showing the ratio of odds of acute side effects: nausea (a) and vomiting (b) (=events) among pediatric patients with brain tumors treated with PT or XRT. OR: odds ratio (proton/photon), CI: confidence interval.

Among the acute side effects, only nausea, vomiting, and skin disorders were reported in at least three articles. The reported side effects were grade 3 or higher. Reduction in nausea was observed [35,37,49,54] in patients receiving PT (OR: 0.30, 95% CI: 0.11–0.78, *I*^*2*^: 0%, *p*: 0.028) versus XRT; however, there no statistically significant difference was observed for vomiting [49,54,55](OR: 0.37, 95% CI: 0.03–4.97, *I*^*2*^*:* 37%, *p*: 0.243) (Fig 5).

Based on skin disorder there was statistical no difference between the radiotherapy methods (OR: 0.69, 95% CI: 0.13–3.80, *I*^*2*^:0%, *p*: 0.453) [59].

Data about the ototoxicity was found in three articles, and based on the statistical analysis there was no difference between the radiotherapy methods (OR:0.78, 95% CI: 0.40–1.52, *I*^*2*^*:* 0%, *p*: 0.249) [23,42,59]. (S9 Fig in S1 File).

The appearance of neurological side effects (OR: 0.78, 95% CI: 0.09–6.90, *I*^*2*^:43%, *p*: 0.744) [49,56,58,59] and ophthalmologic disorders (OR: 0,96, 95% CI: 0.05–17.45, *I*^*2*^: 57%, *p*: 0.971) [46,49,58,59] was not significantly different between the two radiotherapy modalities (S10, S11 Figs in S1 File).

### Hematological side effects

**Fig 6:**
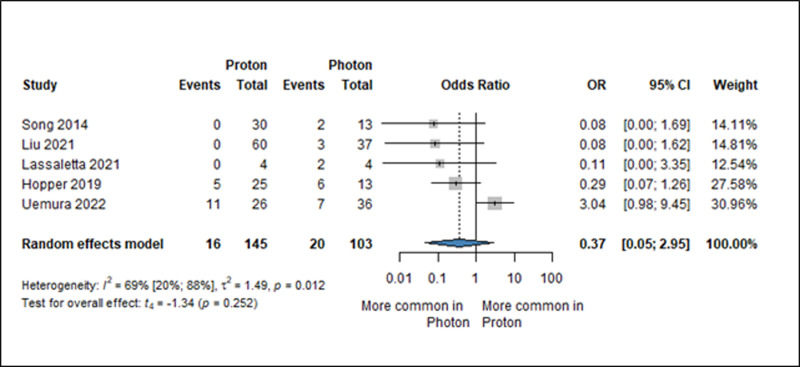
Forest plot showing the ratio of odds of grade 3 anemia (=event) among pediatric patients with brain tumors treated with either PT or XRT. OR: odds ratio (proton/photon), CI: confidence interval.

For hematological adverse events, grade 3 and 4 side effects were analyzed. In the majority of articles concomitant chemotherapy was administered based on personal protocol. For grade 3 anemia, five articles were included in the analysis [35,38,49,54,55], (OR: 0.37, 95% CI: 0.05–2.95, *I*^*2*^: 69%, p: 0.252) (Fig 6). Interestingly, grade 3 leukopenia (OR: 1.24, 95% CI: 0.31–4.86, *I*^*2*^: 52%, p: 0.657) was observed less frequently in XRT as opposed to grade 4 leukopenia (OR:0.35, 95% CI: 0.06–1.96, *I*^*2*^: 0%, p: 0121). When grade 3 thrombocytopenia was considered (OR: 0.55, 95% CI: 0.50–0.61, *I*^*2*^: 0%, p: 0.002), there was a statistically significant difference in favor of PT. For thrombocytopenia grade 4 (OR: 0.61, 95% CI: 0.03–11.84, *I*^*2*^: 35%, p: 0.549), the tendency was that PT could be the better choice, without statistically significant difference. (S12, S13, S14, S15, and S16 Figs in S1 File).

### Risk of bias assessment and quality of evidence

Based on the overall risk of bias assessment, nine articles [21,24,35,43,44,47,61–63] were identified as severe, 14 as moderate [22,23,25,38,42,45,46,49–51,54,57,59,60], and 10 as low risk of bias [35,36,39,41,48,51,52,55,56,58]. The results of the risk of bias assessment are presented in S3 Table in S1 File.

In terms of quality of evidence, all our results had moderate or serious risk of bias. A summary of the findings table can be found in the Supplementary Material (S4 Table in S1 File).

### Publication bias and heterogeneity

We included less than 10 articles per outcome, which is why Egger’s test was not performed. Generally, we have a homogeneous population. In most of the analyses, moderate heterogeneity was observed. This can be due to different study populations, follow-up times, or follow-up protocols used.

## Discussion

This is the first meta-analysis to compare the efficacy and side-effect profile of PT and XRT in children with various types of brain tumors. Only direct comparative studies were included.

Our study found no significant difference between XRT and PT in terms of 5-year overall survival, but there was a significant difference in terms of the incidence of side effects. More children survive cancer treatment each year; thus, to improve their quality of life, the focus should be on minimizing the side effects. A Swedish study found that PT had high potential importance, and it was estimated that 80–100 children could benefit from the therapy each year [64]. In a recent study, where adult medulloblastoma patients were compared based on the used radiotherapy method, found that there is a dosimetric improvement, leading to decreased acute side effects [65].

In this study, the population analyzed received radiotherapy as part of a predefined treatment protocol, and those who received radiotherapy for secondary malignancies were excluded. In a previous meta-analysis, the incidence of secondary malignancies was similar in the two radiotherapy modalities [66] but studies by Xiang and Ludmir et al. found that the incidence of secondary malignancies was lower in PT [11,67]. In another study, there was no difference in the incidence of secondary malignancies between the two radiotherapy modalities [68]. There was also no difference in the incidence of radiation-induced cavernomas between the two radiotherapy modalities [69].

The indications for PT and XRT may be different based on the tumor type. In the studies included, no differences were identified by tumor type or age, all results were published based on overall findings [70].

The 5-year overall survival from the articles and 3,6,9-year data from curves showed that both radiotherapy modalities were safe, demonstrating favorable outcomes. Since PT reduces late adverse events, PT can potentially lead to a better survival outcome over a longer period than XRT, PT can deliver the desired target dose more precisely to the target area, however, the results show the contrary regarding overall survival rate. While the overall survival results show that both are safe with time, with more studies the result can be reversed.

We analyzed the most frequent endocrine side effects. In hypothyroidism, growth hormone, and sex hormone deficiency, we observed a clear effect on the plots. We obtained data from two articles on adrenal insufficiency [45,51] and precocious puberty [51], stating that PT was beneficial in reducing this endocrine side effects as well.

Different, age adapted scales were used to assess the level of decline in the neurocognitive changes group. All articles included mentioned that these scales correlated with each other and could be pooled together, that is why mean difference was used as measure of effect. We are aware that the neurocognitive decline can be affected by radiation dose and target area as well, but there is no clear distinction regarding radiation dose variations. All the domains measured showed that PT was more likely to result in higher neurocognitive performance on these scales compared to XRT. A previous meta-analysis, where neurocognitive outcomes were measured from the same articles as in our study, also concluded that patients who received PT scored higher on neurocognitive outcomes [48]. In three systematic reviews, the main conclusions were the same as in our case: there is a need to use and establish proton centers to reduce neurocognitive side effects [55,71,72].

We collected data on the incidence of acute side effects such as nausea, vomiting, dysphagia, anorexia, diarrhea, constipation, fatigue, headache, insomnia, esophagitis, abdominal pain, neurological disorders, ophthalmologic, and skin disorders. Data on nausea, vomiting, neurological side effects, and ophthalmologic disorders were pooled together because of the lack of data in the articles. Our results showed that PT could reduce the incidence of acute side effects. Results regarding ophthalmologic side effects are similar to a study from 2022, where radiotherapy was compared in the case of optic pathway gliomas [73]. Neurological side effects included ataxia, cranial nerve disturbance, weakness, seizures, dysarthria, somnolence, balance disturbance, and speech problems. Measurement of the effects showed that there was no difference between the two radiotherapy modalities, but there was a tendency for PT to have a beneficial effect in reducing these side effects.

Hematological side effects are more common after chemotherapy; however, radiotherapy can also facilitate their development. These side effects were graded according to the CTC-AE scale, version 4 or 5. Clinically, the most important grades are grades 3 and 4. By the investigation of grade 4 anemia, three articles [38,49,54] provided data. Two of them [38,49] described 0 events; however, in the third [54], half of the patients developed grade 4 anemia in the proton cohort and 1/3 in the photon cohort, which could be due to the different checkup protocols. No other causes could be identified during data collection. In this analysis, PT were more useful as a protective therapy, considering hematological toxicity.

Ototoxicity is a very common side effect in the case of radiotherapy; however, a few studies were identified with appropriate data reporting for this outcome. A retrospective study [42] showed that the use of PT resulted in low early high-grade ototoxicity, as in our results.

Event-free survival was not available in the articles, only progression-free, recurrence-free, and disease-free survival, but none was available in at least three articles.

Based on our study, the overall well-being of survivors can be increased by using PT. In addition, a previously published systematic review, where articles with non-CNS tumors were also included, showed the same findings, as in case of studies including pediatric and adult medulloblastoma patients as well [65,70,72]. With a running multicenter cohort study, where the authors would like to highlight the difference of second cancer risk as well, the French radiotherapy guide and our study, it can be changed the management of radiotherapy among pediatric brain cancer patients, and also a protocol can be designed for a better treatment plan with a high quality of life after treatment [74,75].

Even though some of our results were statistically not significant, there is considerable clinical relevance in the findings which may facilitate the development of protocols regarding the usage of radiotherapy methods, and may encourage the establishment of more proton centers, where more studies can be done.

### Strengths and limitations

Regarding the strengths of our analysis, we followed our protocol, which was registered in advance. A rigorous methodology was subsequently applied. In addition, this is the first comprehensive meta-analysis where multiple outcomes were assessed.

Considering the limitations of this study, we included a small number of articles in our study. The follow-up period was different in the articles, and different types of tumors were analyzed, most of which were medulloblastomas. The presence of moderate and high risks of bias in some of the domains is another limitation. In the case of neurocognitive decline, test results before the radiotherapy were not reported, only those from the last checkup; therefore, the change from baseline could not be assessed. Another limitation is the geographical distribution of the reported studies.

### Implication for practice and research

Based on our results, we suggest conducting more two-arm comparative high-quality prospective studies with longer follow-up periods to detect late side effects. This approach will help determine the best treatment strategy. For neurocognitive decline, reporting initial test results can enhance the study quality by allowing proper assessment of changes from baseline. Translational science is vital for closing the gap between clinical research and its application in everyday medical practice [76,77], This systematic and comprehensive assessment contributes to this effort by analyzing data on proton and photon therapies in pediatric brain cancer patients.

## Conclusion

PT can be beneficial in reducing postradiotherapy toxicity for children with brain tumors without significantly decreasing survival rate. For this, the implementation of more proton centers and an exchange in the protocol is needed. With this radiotherapy method the life of children after radiotherapy can be improved, and by implementing more centers higher quality studies, with more participants can be conducted in this field, so we could better investigate the efficacy of this promising radiotherapy method.

## Key results

1.All the included retrospective cohort studies show us that there is statistically no difference regarding survival (OR: 0.80, 95% CI: 0.51–1.23) in proton or photon-treated patient groups.

2.The frequency of side effects is lower in proton radiotherapy groups as in photon ones, especially in case of hypothyroidism (OR: 0.17, 95% CI: 0.11–0.28), thrombocytopenia grade 3 (OR: 0.55, 95% CI: 0.50–0.61) and nausea (OR: 0.30, 95% CI: 0.11–0.78).

3.Neurocognitive domains, for example IQ level, can be increased by using proton radiotherapy at children with brain cancers (MD: 11.91, 95% CI: 1.91–21.90).

## Supporting information

S1 File Supplementary_Material_31-JAN-2025. (DOCX)

S2 Flie PRISMA_2020_Flow_Diagram_31-JAN-2025. (DOCX)

S3 File List_of_studies_identified_31-JAN-2025. (XLSX)

S4 File Data_table_with_all_identified_articles_31-JAN-2025. (XLSX)

S5 File Data_extraction_table_31-JAN-2025. (XLSX)
