# Supplementary material for: Proton or photon? Comparison of survival and toxicity of two radiotherapy modalities among pediatric brain cancer patients: A systematic review and meta-analysis
Source: PLoS One. 2025 Feb 20;20(2):e0318194. doi: 10.1371/journal.pone.0318194 (PMC11841876; doi:10.1371/journal.pone.0318194)
Supplement: S1 File — (DOCX) [file pone.0318194.s001.docx]

**Supplementary Material**

Proton or photon? Comparison of survival and toxicity of two radiotherapy modalities among pediatric brain cancer patients: a systematic review and meta-analysis

Renáta Kiss-Miki^1,2^, Vanda Máté^1,3^, Mahmoud Obeidat^1^, Brigitta Teutsch^1,4^, Gergely Agócs^1^, Szilvia Kiss-Dala^1^, Péter Hegyi^1, 5^, Janka Kovács^1,3^, Andrea Párniczky^1,6^, Eszter Tuboly^1,7^, Miklós Garami^1,3*^

^1^ Centre for Translational Medicine, Semmelweis University, Budapest, Hungary

^2^ Neurosurgery and Neurointerventional Clinic, Semmelweis University, Budapest, Hungary

^3^ Pediatric Center, Semmelweis University, Budapest, Hungary

^4^ Institute for Translational Medicine, Medical School, University of Pécs, Pécs, Hungary

^5^ Institute of Pancreatic Diseases, Semmelweis University, Budapest, Hungary

^6^ Heim Pál National Pediatric Institute, Budapest, Hungary

^7^ Hungarian Pediatric Oncology Network, Budapest, Hungary

^*^ Corresponding author

E-mail: [garami.miklos@semmelweis.hu](mailto:garami.miklos@semmelweis.hu) (MG)

**Table legends**

**S1 Table. PRISMA checklist.**

**S2 Table. Detailed search key in different databases.**

**S3 Table. The overall risk of bias for all outcomes among analysis.**

**S4 Table. Summary of findings table of the quality of evidence of the investigated outcomes.**

**Figure legends**

**S1 Fig. The 3-year overall survival probability among patients who received PT or RT.** In subgroup 2 are those patients who received PT and in subgroup 1 those, who received RT.

**S2 Fig. The 6-year survival probability among children who received PT or RT.** In subgroup 2 are those patients who received PT and in subgroup 1 those, who received RT.

**S3 Fig. The 9-year survival probability among children who received PT or RT.** In subgroup 2 are those patients who received PT and in subgroup 1 those, who received RT.

**S4 Fig. Growth hormone deficiency among children with brain tumors who received PT or RT.**

**S5 Fig. Sex hormone deficiency among children with brain tumors who received PT or RT.**

**S6 Fig. Forest plot representing the mean difference of working memory among pediatric brain cancer patients receiving PT or RT.**

**S7 Fig. Forest plot representing the mean difference of processing speed among pediatric brain cancer patients receiving PT or RT.**

**S8 Fig. Forest plot representing the mean difference of perceptual reasoning among pediatric brain cancer patients receiving PT or RT.**

**S9 Fig. Forest plot representing the ratio of odds of ototoxicity.**

**S10 Fig. Forest plot representing the ratio of odds of neurological side effects.**

**Forest plot representing the ratio of odds of ophthalmological side effects among pediatric brain cancer patients receiving PT or RT.**

**S12 Fig. Forest plot representing the ratio of odds of anemia grade 4 among pediatric brain cancer patients receiving PT or RT.**

**S13 Fig. Forest plot representing the ratio of odds of leukopenia grade 3 among pediatric brain cancer patients receiving PT or RT.**

**S14 Fig. Forest plot representing the ratio of odds of leukopenia grade 4 among pediatric brain cancer patients receiving PT or RT.**

**S15 Fig. Forest plot representing the ratio of odds of thrombocytopenia grade 3 among pediatric brain cancer patients receiving PT or RT.**

**S16 Fig. Forest plot representing the ratio of odds of thrombocytopenia grade 4 among pediatric brain cancer patients receiving PT or RT.**

**S1 Table**. PRISMA checklist.

| **Section and Topic** | **Item #** | **Checklist item** | **Paper 1 pg.** |
| --- | --- | --- | --- |
| **TITLE** | | |  |
| Title | 1 | Identify the report as a systematic review. | 1 |
| **ABSTRACT** | | |  |
| Abstract | 2 | See the PRISMA 2020 for Abstracts checklist. | 3 |
| **INTRODUCTION** | | |  |
| Rationale | 3 | Describe the rationale for the review in the context of existing knowledge. | 5 |
| Objectives | 4 | Provide an explicit statement of the objective(s) or question(s) the review addresses. | 5 |
| **METHODS** | | |  |
| Eligibility criteria | 5 | Specify the inclusion and exclusion criteria for the review and how studies were grouped for the syntheses. | 6 |
| Information sources | 6 | Specify all databases, registers, websites, organisations, reference lists and other sources searched or consulted to identify studies. Specify the date when each source was last searched or consulted. | 6 |
| Search strategy | 7 | Present the full search strategies for all databases, registers and websites, including any filters and limits used. | Table S2 |
| Selection process | 8 | Specify the methods used to decide whether a study met the inclusion criteria of the review, including how many reviewers screened each record and each report retrieved, whether they worked independently, and if applicable, details of automation tools used in the process. | 6 |
| Data collection process | 9 | Specify the methods used to collect data from reports, including how many reviewers collected data from each report, whether they worked independently, any processes for obtaining or confirming data from study investigators, and if applicable, details of automation tools used in the process. | 6-7 |
| Data items | 10a | List and define all outcomes for which data were sought. Specify whether all results that were compatible with each outcome domain in each study were sought (e.g. for all measures, time points, analyses), and if not, the methods used to decide which results to collect. | 7 |
|  | 10b | List and define all other variables for which data were sought (e.g. participant and intervention characteristics, funding sources). Describe any assumptions made about any missing or unclear information. | 7 |
| Study risk of bias assessment | 11 | Specify the methods used to assess risk of bias in the included studies, including details of the tool(s) used, how many reviewers assessed each study and whether they worked independently, and if applicable, details of automation tools used in the process. | 7 |
| Effect measures | 12 | Specify for each outcome the effect measure(s) (e.g. risk ratio, mean difference) used in the synthesis or presentation of results. | 7 |
| Synthesis methods | 13a | Describe the processes used to decide which studies were eligible for each synthesis (e.g. tabulating the study intervention characteristics and comparing against the planned groups for each synthesis (item #5)). | 7-8 |
|  | 13b | Describe any methods required to prepare the data for presentation or synthesis, such as handling of missing summary statistics, or data conversions. | 7-8 |
|  | 13c | Describe any methods used to tabulate or visually display results of individual studies and syntheses. | 7-8 |
|  | 13d | Describe any methods used to synthesize results and provide a rationale for the choice(s). If meta-analysis was performed, describe the model(s), method(s) to identify the presence and extent of statistical heterogeneity, and software package(s) used. | 7-8 |
|  | 13e | Describe any methods used to explore possible causes of heterogeneity among study results (e.g. subgroup analysis, meta-regression). | 7-8 |
|  | 13f | Describe any sensitivity analyses conducted to assess robustness of the synthesized results. | 8 |
| Reporting bias assessment | 14 | Describe any methods used to assess risk of bias due to missing results in a synthesis (arising from reporting biases). | 7 |
| Certainty assessment | 15 | Describe any methods used to assess certainty (or confidence) in the body of evidence for an outcome. | Table S2 |
| **RESULTS** | | |  |
| Study selection | 16a | Describe the results of the search and selection process, from the number of records identified in the search to the number of studies included in the review, ideally using a flow diagram. | 9 |
|  | 16b | Cite studies that might appear to meet the inclusion criteria, but which were excluded, and explain why they were excluded. | 9 |
| Study characteristics | 17 | Cite each included study and present its characteristics. | 9-10 |
| Risk of bias in studies | 18 | Present assessments of risk of bias for each included study. | 10 |
| Results of individual studies | 19 | For all outcomes, present, for each study: (a) summary statistics for each group (where appropriate) and (b) an effect estimate and its precision (e.g. confidence/credible interval), ideally using structured tables or plots. | 9-10 |
| Results of syntheses | 20a | For each synthesis, briefly summarise the characteristics and risk of bias among contributing studies. | 9-10 |
|  | 20b | Present results of all statistical syntheses conducted. If meta-analysis was done, present for each the summary estimate and its precision (e.g. confidence/credible interval) and measures of statistical heterogeneity. If comparing groups, describe the direction of the effect. | 9-10 |
|  | 20c | Present results of all investigations of possible causes of heterogeneity among study results. | 10 |
|  | 20d | Present results of all sensitivity analyses conducted to assess the robustness of the synthesized results. | 10 |
| Reporting biases | 21 | Present assessments of risk of bias due to missing results (arising from reporting biases) for each synthesis assessed. | 10 |
| Certainty of evidence | 22 | Present assessments of certainty (or confidence) in the body of evidence for each outcome assessed. | Table S2 |
| **DISCUSSION** | | |  |
| Discussion | 23a | Provide a general interpretation of the results in the context of other evidence. | 11 |
|  | 23b | Discuss any limitations of the evidence included in the review. | 12 |
|  | 23c | Discuss any limitations of the review processes used. | 12 |
|  | 23d | Discuss implications of the results for practice, policy, and future research. | 12 |
| **OTHER INFORMATION** | | |  |
| Registration and protocol | 24a | Provide registration information for the review, including register name and registration number, or state that the review was not registered. | 6 |
|  | 24b | Indicate where the review protocol can be accessed, or state that a protocol was not prepared. | 6 |
|  | 24c | Describe and explain any amendments to information provided at registration or in the protocol. | 6 |
| Support | 25 | Describe sources of financial or non-financial support for the review, and the role of the funders or sponsors in the review. | 13 |
| Competing interests | 26 | Declare any competing interests of review authors. | 13 |
| Availability of data, code and other materials | 27 | Report which of the following are publicly available and where they can be found: template data collection forms; data extracted from included studies; data used for all analyses; analytic code; any other materials used in the review. | 15-21, Supplementary material |

**S2 Table**. Detailed search key in different databases.

| **Database** | **Searchkey** |
| --- | --- |
| PubMed | **Pediatric**: (**pediatric*** OR paediatric* OR adolescent OR adolescence OR child* OR kids OR kid OR youth OR juvenile OR infant* OR infancy OR preschooler* OR teen OR teens OR teenager*)  *AND*  **Brain cancer**: (astrocytoma OR glioblastoma OR DIPG OR “glioma” OR “HGG” OR “LGG” OR “ATRT” OR “PNET” OR medulloblastoma OR dysgerminoma OR oligodendroglioma OR xanthoastrocytoma OR astroblastoma OR ganglioglioma OR gangliocytoma OR “Lhermitte-Duclos disease“ OR “neurocytoma“ OR “cerebellar liponeurocytoma“ OR ependymoma OR subependymoma OR “choroid plexus papilloma” OR pineocytoma OR pineoblastoma OR schwannoma OR neurofibroma OR perineurioma OR paraganglioma OR meningeoma OR “CNS hemangioma“ OR “CNS vascular malformation“ OR “meningeal melanocytosis“ OR “meningeal melanomatosis“ OR “meningeal melanocytoma“ OR germinoma OR choriocarcinoma OR craniopharyngeoma OR pituicytoma OR oncocytoma OR “pituitary adenoma“ OR “PitNET“ OR “pituitary blastoma“ OR “**brain cancer**“ OR “brain cancers“ OR “central nervous system tumor” OR “central nervous system tumour” OR “brain malignoma”)  *AND*  **Radiotherapy**: proton AND (**photon*** OR “x-ray radiotherapy“ OR “intensity modulated radiotherapy“ OR “Cyberknife“ OR “Gamma knife“ OR “stereotactic radiotherapy“ OR “stereotactic radiosurgery“ OR “3d conformal radiotherapy“ OR “volumetric modulated arch therapy“ OR “particle beam therapy“ OR “image guided radiotherapy“ OR radiation* OR irradiation*) |
| Embase | **Pediatric**: (**pediatric*** OR paediatric* OR adolescent OR adolescence OR child* OR kids OR kid OR youth OR juvenile OR infant* OR infancy OR preschooler* OR teen OR teens OR teenager*)  *AND*  **Brain cancer**: (astrocytoma OR glioblastoma OR DIPG OR “glioma” OR “HGG” OR “LGG” OR “ATRT” OR “PNET” OR medulloblastoma OR dysgerminoma OR oligodendroglioma OR xanthoastrocytoma OR astroblastoma OR ganglioglioma OR gangliocytoma OR “Lhermitte-Duclos disease“ OR “neurocytoma“ OR “cerebellar liponeurocytoma“ OR ependymoma OR subependymoma OR “choroid plexus papilloma” OR pineocytoma OR pineoblastoma OR schwannoma OR neurofibroma OR perineurioma OR paraganglioma OR meningeoma OR “CNS hemangioma“ OR “CNS vascular malformation“ OR “meningeal melanocytosis“ OR “meningeal melanomatosis“ OR “meningeal melanocytoma“ OR germinoma OR choriocarcinoma OR craniopharyngeoma OR pituicytoma OR oncocytoma OR “pituitary adenoma“ OR “PitNET“ OR “pituitary blastoma“ OR “**brain cancer**“ OR “brain cancers“ OR “central nervous system tumor” OR “central nervous system tumour” OR “brain malignoma”)  *AND*  **Radiotherapy**: proton AND (**photon*** OR “x-ray radiotherapy“OR “intensity modulated radiotherapy“ OR “Cyberknife“ OR “Gamma knife“ OR “stereotactic radiotherapy“ OR “stereotactic radiosurgery“ OR “3d conformal radiotherapy“ OR “volumetric modulated arch therapy“ OR “particle beam therapy“ OR “image guided radiotherapy“ OR radiation* OR irradiation*) |
| Cohrane Library | **Pediatric**: (**pediatric*** OR paediatric* OR adolescent OR adolescence OR child* OR kids OR kid OR youth OR juvenile OR infant* OR infancy OR preschooler* OR teen OR teens OR teenager*)  *AND*  **Brain cancer**: (astrocytoma OR glioblastoma OR DIPG OR “glioma” OR “HGG” OR “LGG” OR “ATRT” OR “PNET” OR medulloblastoma OR dysgerminoma OR oligodendroglioma OR xanthoastrocytoma OR astroblastoma OR ganglioglioma OR gangliocytoma OR “Lhermitte-Duclos disease“ OR “neurocytoma“ OR “cerebellar liponeurocytoma“ OR ependymoma OR subependymoma OR “choroid plexus papilloma” OR pineocytoma OR pineoblastoma OR schwannoma OR neurofibroma OR perineurioma OR paraganglioma OR meningeoma OR “CNS hemangioma“ OR “CNS vascular malformation“ OR “meningeal melanocytosis“ OR “meningeal melanomatosis“ OR “meningeal melanocytoma“ OR germinoma OR choriocarcinoma OR craniopharyngeoma OR pituicytoma OR oncocytoma OR “pituitary adenoma“ OR “PitNET“ OR “pituitary blastoma“ OR “**brain cancer**“ OR “brain cancers“ OR “central nervous system tumor” OR “central nervous system tumour” OR “brain malignoma”)  *AND*  **Radiotherapy**: proton AND (**photon*** OR “x-ray radiotherapy“OR “intensity modulated radiotherapy“ OR “Cyberknife“ OR “Gamma knife“ OR “stereotactic radiotherapy“ OR “stereotactic radiosurgery“ OR “3d conformal radiotherapy“ OR “volumetric modulated arch therapy“ OR “particle beam therapy“ OR “image guided radiotherapy“ OR radiation* OR irradiation*) |
| Scopus | **ediatric**: (**pediatric*** OR paediatric* OR adolescent OR adolescence OR child* OR kids OR kid OR youth OR juvenile OR infant* OR infancy OR preschooler* OR teen OR teens OR teenager*)  *AND*  **Brain cancer**: (astrocytoma OR glioblastoma OR DIPG OR “glioma” OR “HGG” OR “LGG” OR “ATRT” OR “PNET” OR medulloblastoma OR dysgerminoma OR oligodendroglioma OR xanthoastrocytoma OR astroblastoma OR ganglioglioma OR gangliocytoma OR “Lhermitte-Duclos disease“ OR “neurocytoma“ OR “cerebellar liponeurocytoma“ OR ependymoma OR subependymoma OR “choroid plexus papilloma” OR pineocytoma OR pineoblastoma OR schwannoma OR neurofibroma OR perineurioma OR paraganglioma OR meningeoma OR “CNS hemangioma“ OR “CNS vascular malformation“ OR “meningeal melanocytosis“ OR “meningeal melanomatosis“ OR “meningeal melanocytoma“ OR germinoma OR choriocarcinoma OR craniopharyngeoma OR pituicytoma OR oncocytoma OR “pituitary adenoma“ OR “PitNET“ OR “pituitary blastoma“ OR “**brain cancer**“ OR “brain cancers“ OR “central nervous system tumor” OR “central nervous system tumour” OR “brain malignoma”)  *AND*  **Radiotherapy**: proton AND (**photon*** OR “x-ray radiotherapy“OR “intensity modulated radiotherapy“ OR “Cyberknife“ OR “Gamma knife“ OR “stereotactic radiotherapy“ OR “stereotactic radiosurgery“ OR “3d conformal radiotherapy“ OR “volumetric modulated arch therapy“ OR “particle beam therapy“ OR “image guided radiotherapy“ OR radiation* OR irradiation*) |


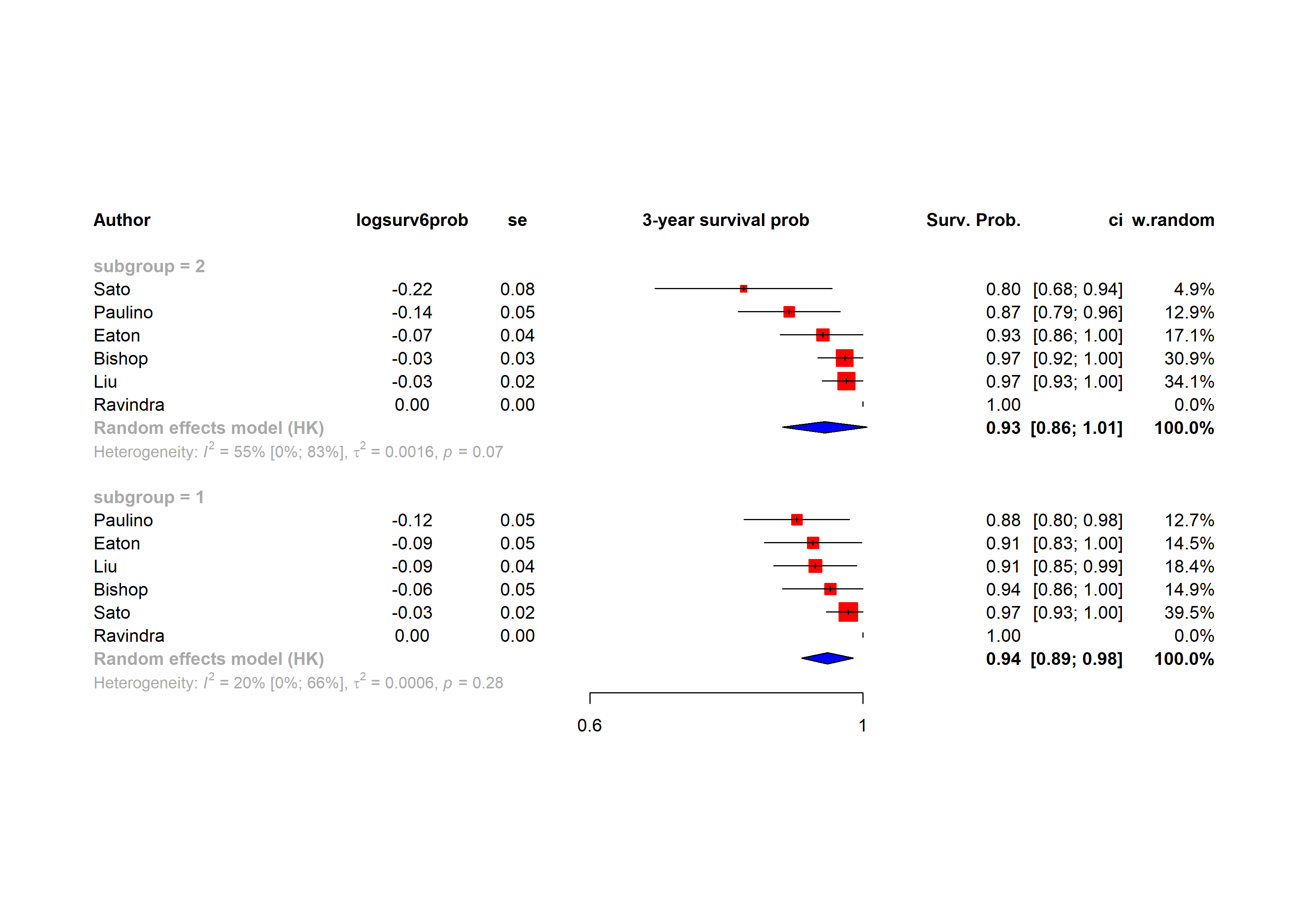


**S1 Fig**. The 3-year overall survival probability among patients who received proton or photon radiotherapy. In subgroup 2 are those patients who received PT and in subgroup 1 those, who received RT.


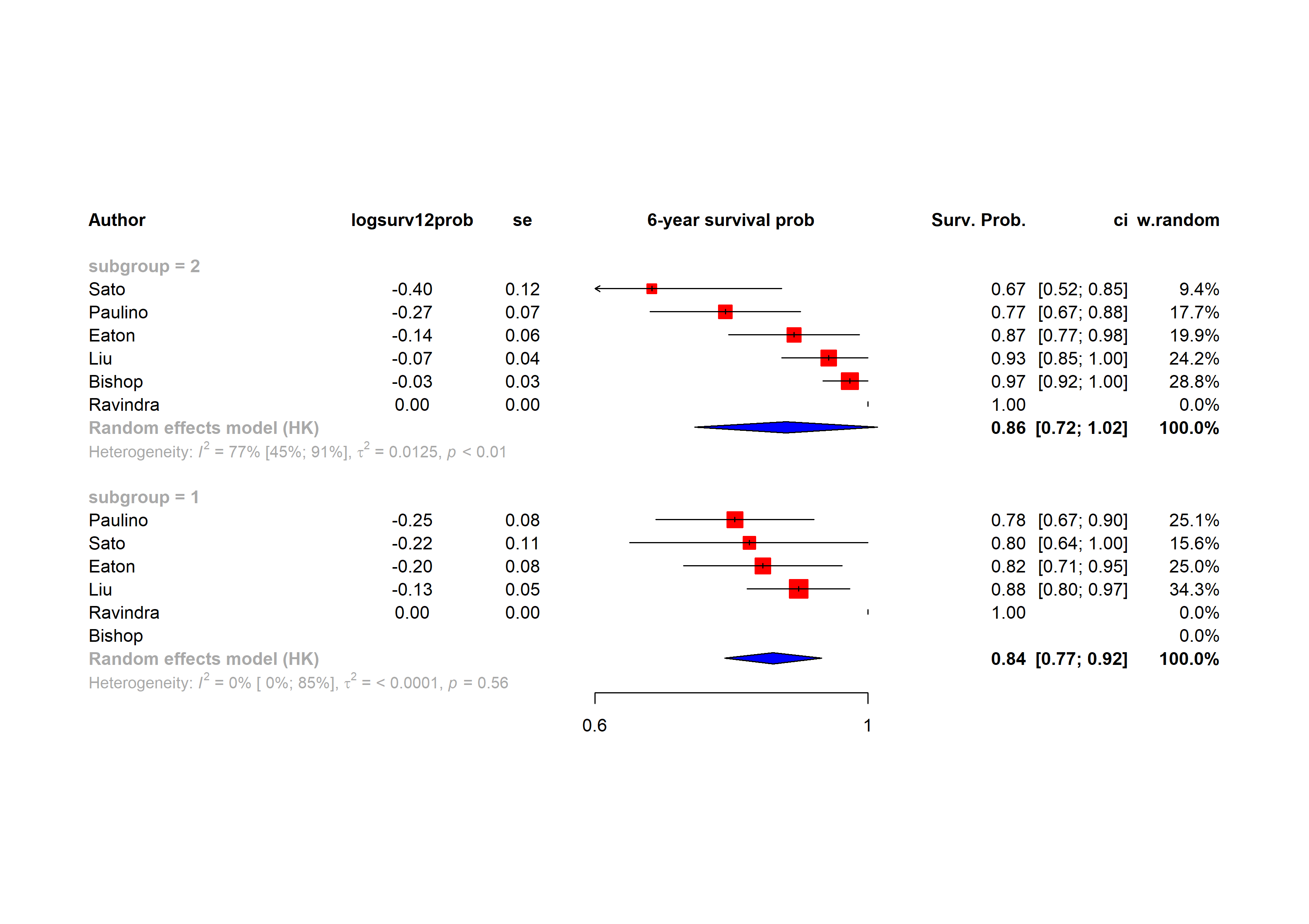


**S2 Fig**. The 6-year survival probability among children who received proton or photon radiotherapy. In subgroup 2 are those patients who received PT and in subgroup 1 those, who received RT.


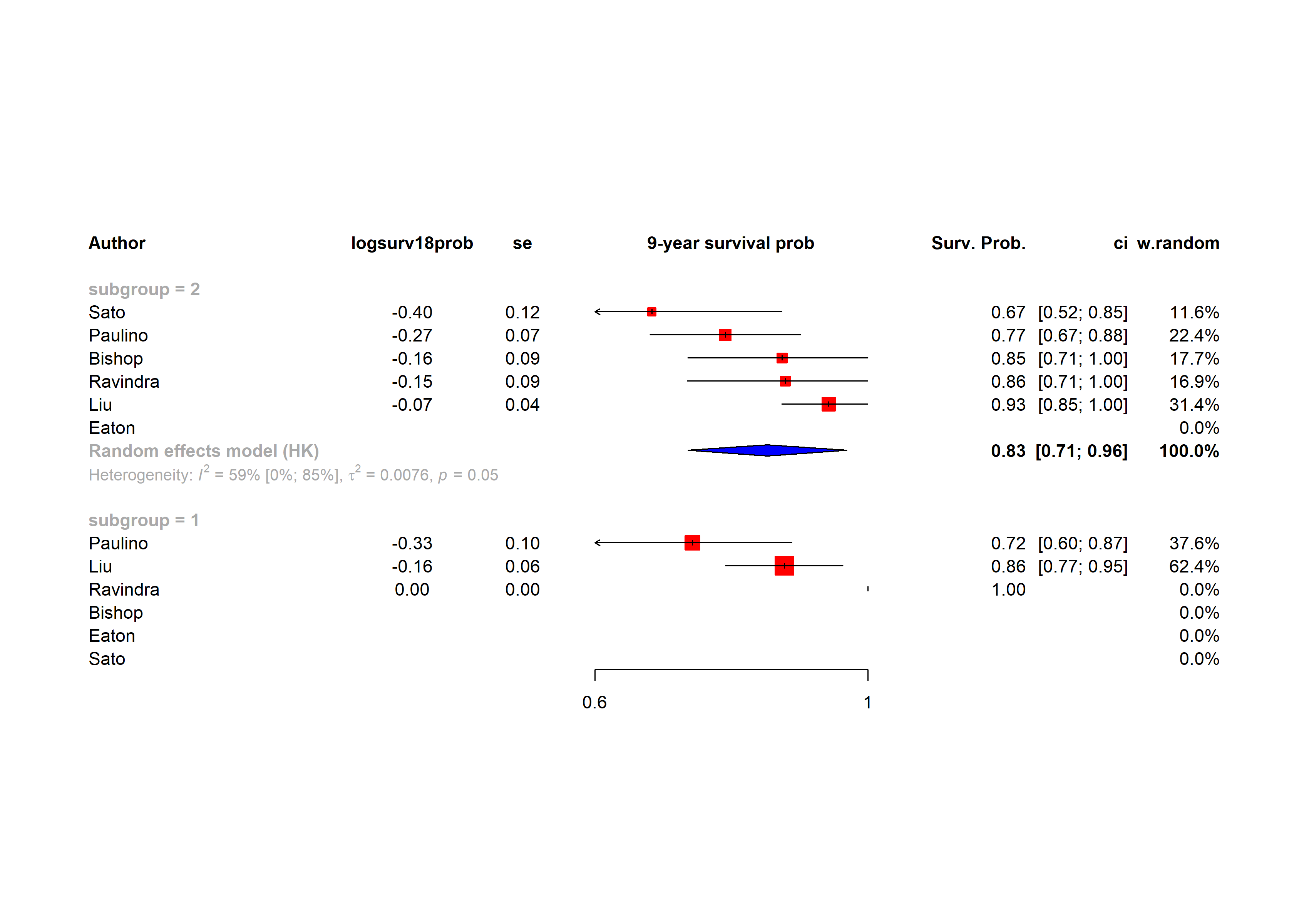


**S3 Fig**. The 9-year survival probability among children who received proton or photon radiotherapy. In subgroup 2 are those patients who received PT and in subgroup 1 those, who received RT.


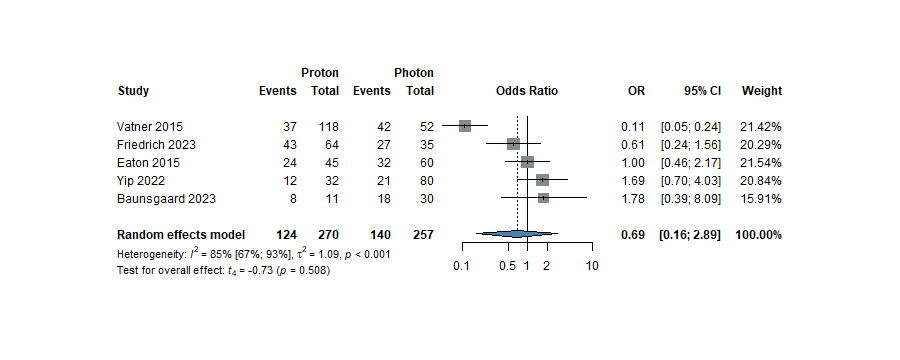


**S4 Fig**. Growth hormone deficiency (events) among children with brain tumors who received PT or RT. OR: odd ratio (proton/photon), 95% CI: confidence interval.


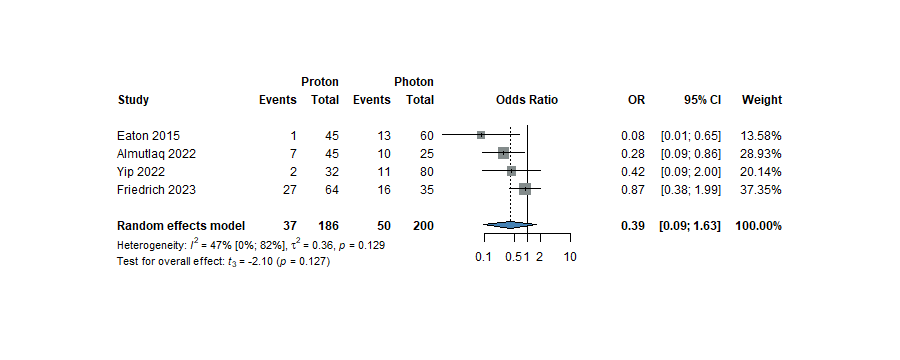


**S5 Fig**. Sex hormone deficiency (events) among children with brain tumors who received PT or RT. OR: odd ratio (proton/photon), 95% CI: confidence interval.


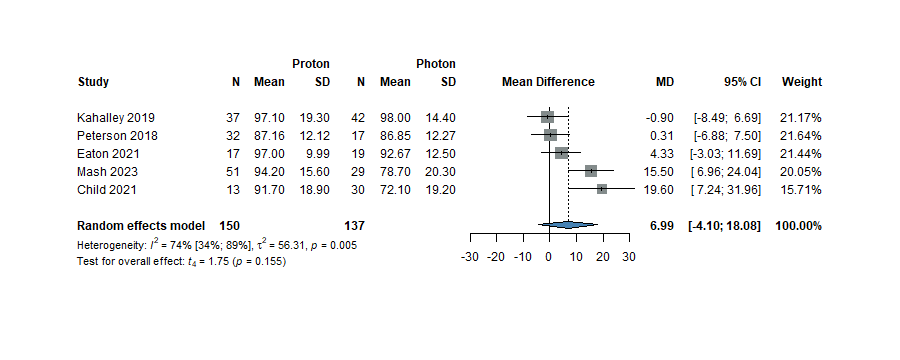


**S6 Fig**. Forest plot representing the mean difference of working memory among pediatric brain cancer patients receiving PT or RT. MD: mean difference, CI: confidence interval.


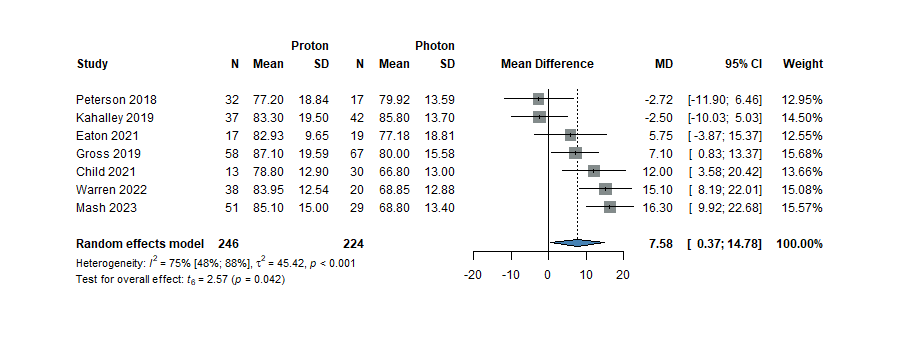


**S7 Fig**. Forest plot representing the mean difference of processing speed among pediatric brain cancer patients receiving PT or RT. MD: mean difference, CI: confidence interval.


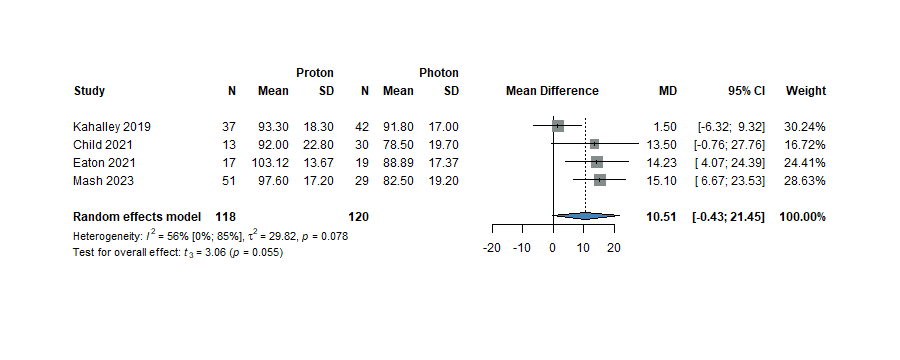


**S8 Fig.**  Forest plot representing the mean difference of perceptual reasoning among pediatric brain cancer patients receiving PT or RT. MD: mean difference, CI: confidence interval.


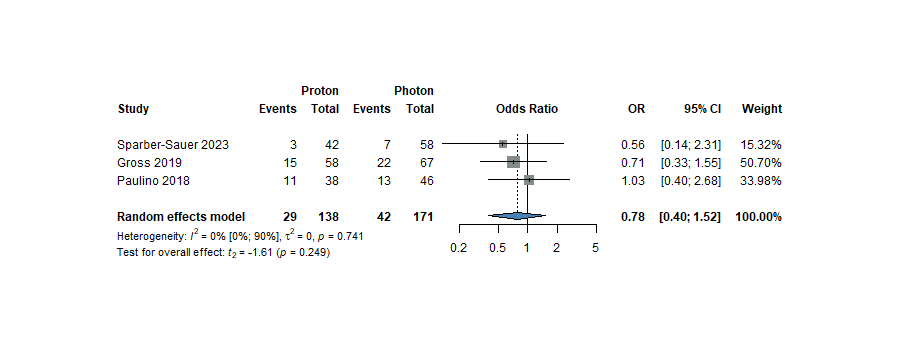


**S9 Fig.** Forest plot representing the odds of ototoxicity among pediatric brain cancer patients receiving PT or RT. OR: odd ratio (proton/photon), CI: confidence interval.


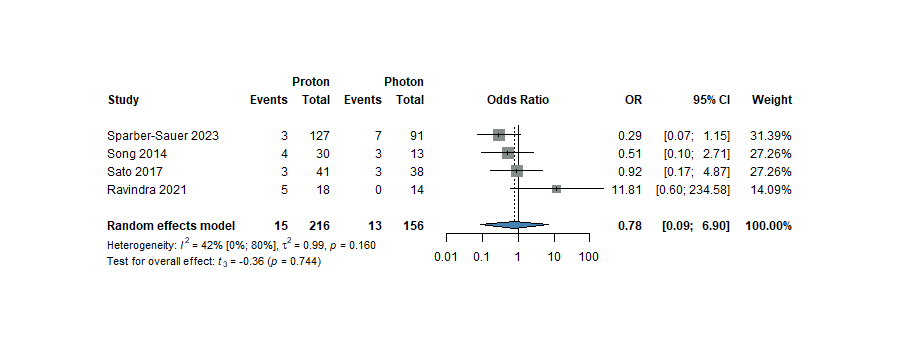


**S10 Fig**. Forest plot representing the odds of **neurological side effects** (events) (ataxia, cranial nerv disturbance, side weakness, seizures, dysarthria, somnolence, balance disturbance, speech problem) among pediatric brain cancer patients receiving PT or RT. OR: odd ratio (proton/photon), CI: confidence interval.


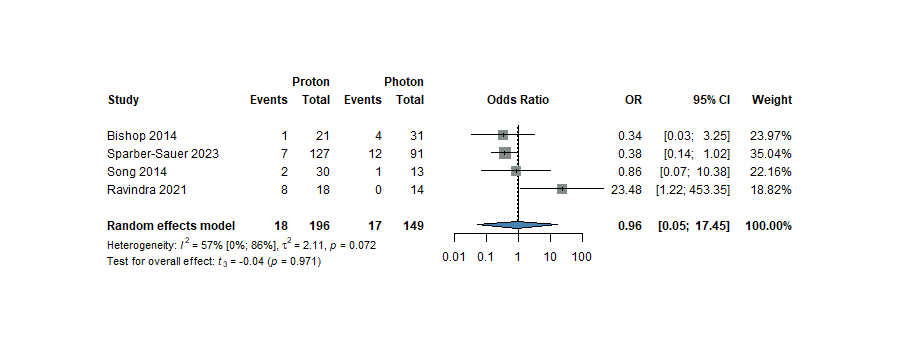


**S11 Fig**. Forest plot representing the ratio of odds of ophthalmological side effects (events) among pediatric brain cancer patients receiving PT or RT. OR: odds ratio (proton/photon), CI: confidence interval.


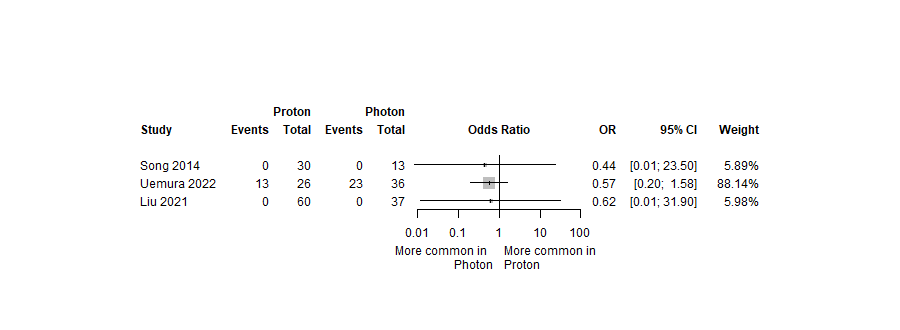


**S12 Fig**. Forest plot representing the ratio of odds of anemia grade 4 (events) among pediatric brain cancer patients receiving PT or RT. OR: odds ratio (proton/photon), CI: confidence interval.


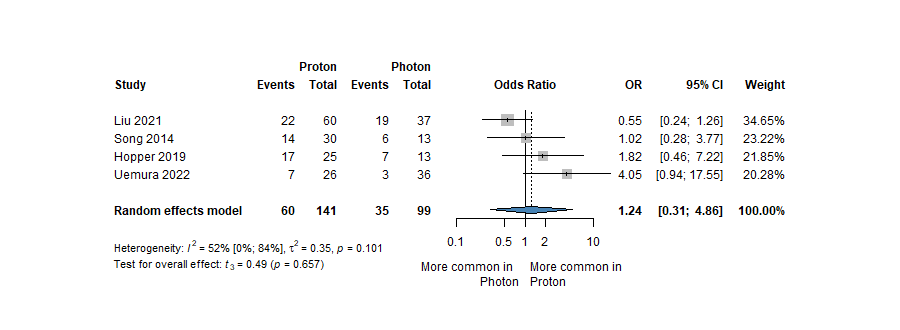


**S13 Fig.** Forest plot representing the ratio of odds of leukopenia grade 3 (events) among pediatric brain cancer patients receiving PT or RT. OR: odds ratio (proton/photon), CI: confidence interval.


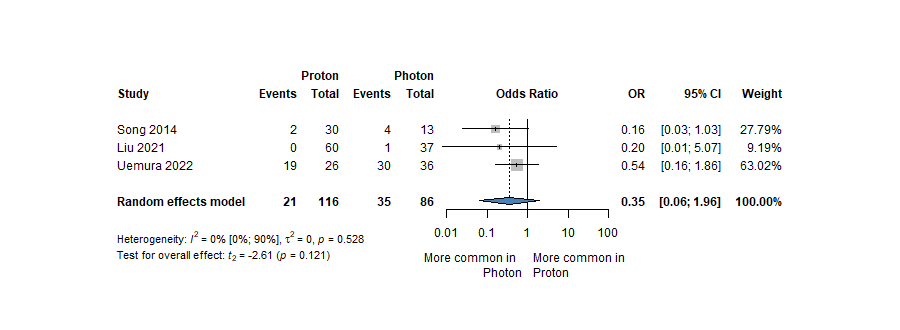


**S14 Fig.** Forest plot representing the ratio of odds of leukopenia grade 4 (events) among pediatric brain cancer patients receiving PT or RT. OR: odds ratio (proton/photon), CI: confidence interval.


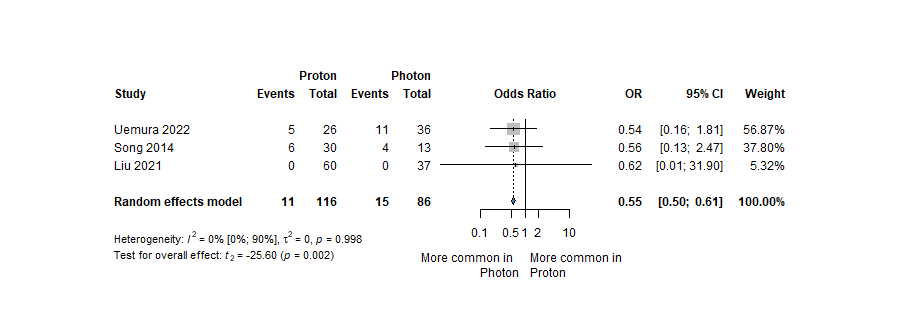


**S15 Fig.** Forest plot representing the ratio of odds of thrombocytopenia grade 3 (events) among pediatric brain cancer patients receiving PT or RT. OR: odds ratio (proton/photon), CI: confidence interval


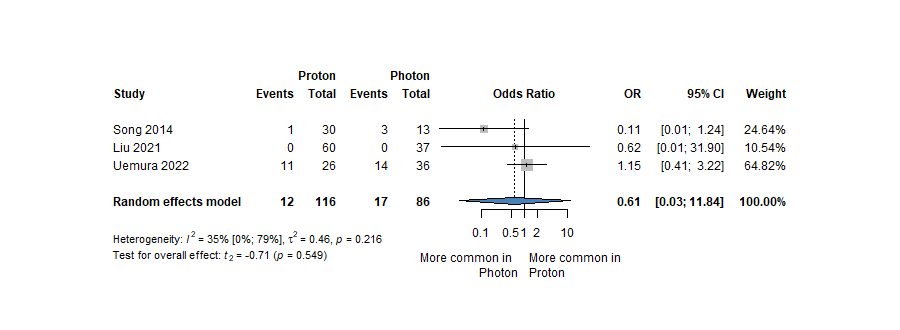


**S16 Fig**. Forest plot representing the ratio of odds of thrombocytopenia grade 4 (events) among pediatric brain cancer patients receiving PT or RT. OR: odds ratio (proton/photon), CI: confidence interval.

**S3 Table.** The overall risk of bias in for all outcomes among analysis.

**
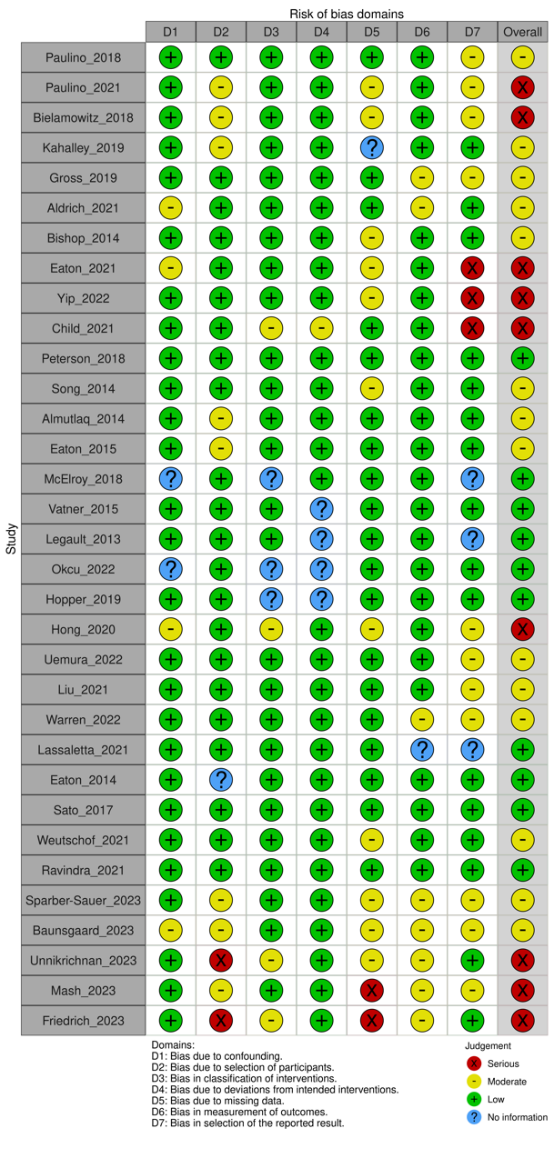
**

**S4 Table**. Summary of findings table of the quality of evidence of the investigated outcomes.

| **Certainty assessment** | | | | | | | **№ of patients** | | **Effect** | | **Certainty** | **Importance** |
| --- | --- | --- | --- | --- | --- | --- | --- | --- | --- | --- | --- | --- |
| **№ of studies** | **Study design** | **Risk of bias** | **Inconsistency** | **Indirectness** | **Imprecision** | **Other considerations** | **proton** | **photon** | **Relative (95% CI)** | **Absolute (95% CI)** |  |  |
| **Hypothyroidism (assessed with: OR)** | | | | | | | | | | | | |
| 8 | non-randomised studies | not serious | not serious | not serious | not serious | all plausible residual confounding would suggest spurious effect, while no effect was observed | 133/478 (27.8%) | 247/459 (53.8%) | **OR 0.22** (0.10 to 0.48) | **334 fewer per 1,000** (from 434 fewer to 179 fewer) | ⨁⨁⨁◯ Moderate | IMPORTANT |
| **Growth hormon defficiency (assessed with: OR)** | | | | | | | | | | | | |
| 3 | non-randomised studies | not serious | not serious | not serious | not serious | all plausible residual confounding would suggest spurious effect, while no effect was observed | 124/270 (45.9%) | 140/257 (54.5%) | **OR 0.69** (0.16 to 2.89) | **92 fewer per 1,000** (from 384 fewer to 231 more) | ⨁⨁⨁◯ Moderate | IMPORTANT |
| **Sex hormon defficiency (assessed with: OR)** | | | | | | | | | | | | |
| 3 | non-randomised studies | serious | not serious | not serious | not serious | publication bias strongly suspected dose response gradient | 37/186 (19.9%) | 50/200 (25.0%) | **OR 0.39** (0.09 to 1.63) | **135 fewer per 1,000** (from 221 fewer to 102 more) | ⨁◯◯◯ Very low | IMPORTANT |
| **Overall survival (assessed with: OR)** | | | | | | | | | | | | |
| 5 | non-randomised studies | not serious | not serious | not serious | serious | publication bias strongly suspected all plausible residual confounding would suggest spurious effect, while no effect was observed | 181/207 (87.4%) | 226/250 (90.4%) | **OR 0.80** (0.51 to 1.23) | **21 fewer per 1,000** (from 76 fewer to 17 more) | ⨁◯◯◯ Very low |  |
| **Anaemia grade 3** | | | | | | | | | | | | |
| 5 | non-randomised studies | not serious | not serious | not serious | not serious | strong association | 16/145 (11.0%) | 20/103 (19.4%) | **OR 0.37** (0.05 to 2.95) | **112 fewer per 1,000** (from 182 fewer to 221 more) | ⨁⨁⨁◯ Moderate | IMPORTANT |
| **Anaemia grade 4** | | | | | | | | | | | | |
| 3 | non-randomised studies | not serious | not serious | not serious | not serious | strong association | 13/116 (11.2%) | 23/86 (26.7%) | **OR 0.56** (0.46 to 0.68) | **98 fewer per 1,000** (from 124 fewer to 69 fewer) | ⨁⨁⨁◯ Moderate | IMPORTANT |
| **Leukopenia grade 3** | | | | | | | | | | | | |
| 4 | non-randomised studies |  |  |  |  |  | 60/141 (42.6%) | 35/99 (35.4%) | **OR 1.24** (0.31 to 4.86) | **51 more per 1,000** (from 209 fewer to 373 more) | - | IMPORTANT |
| **Leukopenia grade 4** | | | | | | | | | | | | |
| 3 | non-randomised studies |  |  |  |  |  | 21/116 (18.1%) | 35/86 (40.7%) | **OR 0.35** (0.06 to 1.96) | **213 fewer per 1,000** (from 367 fewer to 167 more) | - | IMPORTANT |
| **Trombocytopenia grade 3** | | | | | | | | | | | | |
| 3 | non-randomised studies |  |  |  |  |  | 11/116 (9.5%) | 15/86 (17.4%) | **OR 0.55** (0.50 to 0.61) | **70 fewer per 1,000** (from 79 fewer to 60 fewer) | - | IMPORTANT |
| **Trombocytopenia grade 4** | | | | | | | | | | | | |
| 3 | non-randomised studies |  |  |  |  |  | 12/116 (10.3%) | 17/86 (19.8%) | **OR 0.61** (0.03 to 11.84) | **67 fewer per 1,000** (from 190 fewer to 547 more) | - | IMPORTANT |
| **Nausea** | | | | | | | | | | | | |
| 4 | non-randomised studies |  |  |  |  |  | 18/85 (21.2%) | 26/66 (39.4%) | **OR 0.30** (0.11 to 0.78) | **231 fewer per 1,000** (from 327 fewer to 58 fewer) | - | IMPORTANT |
| **Vomiting** | | | | | | | | | | | | |
| 3 | non-randomised studies |  |  |  |  |  | 12/60 (20.0%) | 21/53 (39.6%) | **OR 0.37** (0.03 to 4.97) | **201 fewer per 1,000** (from 377 fewer to 369 more) | - | IMPORTANT |
| **Neurologic disorder** | | | | | | | | | | | | |
| 3 | non-randomised studies |  |  |  |  |  | 15/216 (6.9%) | 13/156 (8.3%) | **OR 0.78** (0.09 to 6.90) | **17 fewer per 1,000** (from 75 fewer to 302 more) | - | IMPORTANT |
| **Ophtalmologic disorder** | | | | | | | | | | | | |
| 3 | non-randomised studies |  |  |  |  |  | 18/196 (9.2%) | 17/149 (11.4%) | **OR 0.96** (0.05 to 17.45) | **4 fewer per 1,000** (from 108 fewer to 578 more) | - | IMPORTANT |
| **IQ level** | | | | | | | | | | | | |
| 5 | non-randomised studies |  |  |  |  |  | 214 | 207 | - | MD **13.06 higher** (4.97 higher to 21.15 higher) | - | IMPORTANT |
| **Working memory** | | | | | | | | | | | | |
| 3 | non-randomised studies |  |  |  |  |  | 150 | 137 | - | MD **6.99 higher** (4.1 lower to 18.08 higher) | - | IMPORTANT |
| **Processing speed** | | | | | | | | | | | | |
| 5 | non-randomised studies |  |  |  |  |  | 246 | 224 | - | MD **7.58 higher** (0.37 higher to 14.78 higher) | - | IMPORTANT |
| **Perceptual reasoning** | | | | | | | | | | | | |
| 3 | non-randomised studies |  |  |  |  |  | 67 | 91 | - | MD **10.51 higher** (0.43 lower to 21.45 higher) | - | IMPORTANT |
| **Ototoxicity** | | | | | | | | | | | | |
| 3 | non-randomised studies |  |  |  |  |  | 29/138 (21.0%) | 0.0% | **OR 0.78** (0.40 to 1.52) | **0 fewer per 1,000** (from 0 fewer to 0 fewer) | - | IMPORTANT |
| **skin disorder** | | | | | | | | | | | | |
| 3 | non-randomised studies |  |  |  |  |  | 15/183 (8.2%) | 12/140 (8.6%) | **OR 0.69** (0.13 to 3.80) | **25 fewer per 1,000** (from 74 fewer to 177 more) | - | IMPORTANT |
