# Supplementary material for: Proton or photon? Comparison of survival and toxicity of two radiotherapy modalities among pediatric brain cancer patients: A systematic review and meta-analysis
Source: PLoS One. 2025 Feb 20;20(2):e0318194. doi: 10.1371/journal.pone.0318194 (PMC11841876; doi:10.1371/journal.pone.0318194)
Supplement: S2 Flie — (DOCX) [file pone.0318194.s002.docx]

**Identification of studies via other methods**

**Identification of studies via databases and registers**

Records identified from:

Websites (n = 0)

Organisations (n = 0)

Citation searching (n = 647)

etc.

Records removed *before screening*:

Duplicate records removed (n = 2119)

Records marked as ineligible by automation tools (n = 0)

Records removed for other reasons (n = 0)

Records identified from*:

Databases (n = 4)

PubMed=1725

Embase=2191

Scopus=818

Cochrane=23

Registers (n = 0)

**Identification**

Records screened

(n = 2639)

Records excluded**

(n = 2534)

Reports not retrieved

(n = 5)

Reports sought for retrieval

(n = 52)

Reports sought for retrieval

(n = 105)

Reports not retrieved

(n = 51)

**Screening**

Reports excluded:

Wrong population (n = 30)

Wrong publication type (n = 8)

Wrong outcome (n = 5)

Wrong study design (n= 3)

Wrong drug (n= 1)

Wrong dosing (n= 2)

Reports excluded:

Wrong population (n = 7)

Wrong publication type (n = 5)

Wrong outcome (n = 4)

Wrong study design (n= 4)

Wrong drug (n= 3)

Wrong dosing (n= 1)

Reports assessed for eligibility

(n = 3)

Reports assessed for eligibility

(n = 54)

Studies included in review

(n = 30)

Reports of included studies from the citation searching

(n = 3)

Total included studies (n= 33)

**Included**
